# Early recovery after endoscopic totally extraperitoneal (TEP) hernia repair in athletes with inguinal disruption: A prospective cohort study

**DOI:** 10.1371/journal.pone.0226011

**Published:** 2019-12-05

**Authors:** Erwin Brans, Inge H. F. Reininga, Hans Balink, Arvid V. E. Munzebrock, Bram Bessem, Joost S. de Graaf

**Affiliations:** 1 Department of Surgery, Medical Center Leeuwarden, Leeuwarden, The Netherlands; 2 University of Groningen, University Medical Center Groningen, Center for Sports Medicine, Groningen, The Netherlands; 3 University of Groningen, University Medical Center Groningen, Department of Trauma Surgery, Groningen, The Netherlands; 4 Department of Nuclear Medicine, Medical Center Leeuwarden, Leeuwarden, The Netherlands; China Medical University Hospital, TAIWAN

## Abstract

**Background:**

Groin pain is a common problem in athletes which results in loss of playing time. Moreover, it can be for the cause of athletic career termination. A common cause of groin pain in athletes is inguinal disruption; pain in the groin area near the pubic tubercle were no obvious other pathology exists to explain the symptoms. Aim of this study was to evaluate the effect of endoscopic totally extraperitoneal (TEP) hernia repair in athletes with inguinal disruption.

**Methods:**

Thirty-one athletes with chronic groin pain due to inguinal disruption, who had undergone conservative therapy without any effect, were included in this prospective cohort study. Prior to surgery patients were assessed by clinical examination, ultrasound of the inguinal region, x-ray and a radionuclide bone scan with single photon-emission computed tomography and CT (SPECT-CT). TEP hernia repair was performed and a lightweight polypropylene mesh was placed pre-peritoneally. Additionally the athletes’ perception about their groin disability was assessed preoperatively and 6 weeks postoperatively by means of the Hip and Groin Outcome Score (HAGOS). The HAGOS consists of six subscales: Pain, Symptoms, Physical function in daily living, Physical function in Sport and Recreation, Participation in Physical Activities, and hip and/or groin-related Quality of Life.

**Results:**

No complications occurred during and after surgery. After six weeks patients improved in all the separate subscales of the Hip and Groin Outcome Score (HAGOS). Within 6 weeks of surgery, 26 patients (84%) returned to sports activities with no or less groin pain.

**Conclusions:**

This study showed that endoscopic totally extraperitoneal (TEP) hernia repair is an effective surgical treatment of inguinal disruption in athletes with chronic groin pain.

## Introduction

Chronic groin pain, commonly seen in athletes, is a debilitating condition which often results in a reduced playing time. Moreover, it may cause the end of a professional athlete’s career. This condition is common in sports involving repetitive kicking movements and rapid directional changes because of the forces exerted around the pelvic area [1,2]. There is a wide variety in terminology that is used to describe this chronic groin pain in athletes, for instance: sportman’s hernia, Gilmore’s groin, groin disruption, pubic inguinal pain syndrome or pubalgia. Therefore, a consensus conference was held by the British Hernia Society in Manchester, UK 2012 [3], followed by the Doha Agreement Meeting on terminology and definitions in groin pain in athletes in Doha, Quatar 2014 [4]. Following both Manchester and Doha statements the term ‘‘inguinal disruption/inguinal related groin pain” was proposed as nomenclature for this condition. The term ‘‘sportman’s hernia” should be rejected, since no actual hernia exist. Inguinal related groin pain, as defined in Doha, describes the location of the pain without explaining its underlying pathology, whereas inguinal disruption, as described in Manchester, defines a specific cause of groin pain related to the inguinal canal. Inguinal disruption was defined as pain which occurs in the groin area where no obvious other pathology exists to explain the symptoms. Pathogenesis of inguinal disruption is multifactorial: posterior wall weakness, superficial inguinal ring dilatation, conjoint tendon tears and damage in the inguinal ligament all have been reported. Multiple underlying pathology may coexist in the athlete with groin pain resulting in an abnormal tension in the inguinal canal [3]. The fact that placement of a mesh to reinforce the posterior wall resolves this problem, supports this [5–7].

No diagnostic imaging modality with high sensitivity and/or specificity to diagnose inguinal disruption is available. Coexisting pathology and symptoms are often present in the groin [8]. It is clear that this frequent overlap of different pathologies causing chronic groin pain often makes the definitive diagnosis, and consequently the appropriate management, difficult and multifactorial. In inguinal disruption, imaging is only used for excluding other pathologies of the hip and/or groin.

Initial treatment usually starts with conservative treatment, consisting of refraining from sport, non-steroidal anti-inflammatory drugs, cortisone injections, shockwave and physiotherapy. Research has shown that physiotherapy should involve an active training program aimed at improving strength and coordination of the muscles around the pelvis and adductors [9,10].

Management of inguinal disruption by surgical exploration and mesh placement should be considered when non-operative treatment fails [6,11,12]. A variety of surgical techniques have been reported in the literature [11,13,14], where laparoscopic surgery tends to have lower pain scores [15] and faster recovery rates compared to open surgery [11,16–19]. However, there is a lack of prospective studies on the effectiveness of operative treatment of groin pain in athletes. Moreover, only one study assessed the patients perspective of recovery following surgical treatment of inguinal disruption [20]. Because groin pain is difficult to assess objectively, the subjective perspective of the athlete may provide valuable information on the severity and impact of groin pain on physical function and quality of life. Therefore, Thorborg et al. [21] developed the Copenhagen Hip and Groin Outcome Score (HAGOS) that can be used to assess function and quality of life in young patients with groin pain.

Hence, aim of this prospective cohort study was to evaluate the early recovery of athletes with inguinal disruption after endoscopic totally extraperitoneal (TEP) hernia repair in terms of physical functioning and quality of life.

## Methods

### Study population

Athletes with undiagnosed chronic groin pain that were referred between 2013 and 2017 to the Medical Center of Leeuwarden, The Netherlands, were eligible for inclusion in the study. Inguinal disruption was defined, according to the Manchester Consensus Conference [3], as pain which occurs in the groin area near the pubic tubercle where no obvious other pathology exists to explain the symptoms. Inguinal disruption was diagnosed if at least three out of the five following clinical signs below are detectable [3]:

1. Pinpoint tenderness over the pubic tubercle at the point of insertion of the conjoint tendon.
2. Palpable tenderness over the deep inguinal ring.
3. Pain and/or dilation of the external ring with no obvious hernia evident.
4. Pain at the origin of the adductor longus tendon.
5. Dull, diffused pain in the groin, often radiating to the perineum and inner thigh or across the midline.

Patients eligible for inclusion were males between 18–65 years old who are limited in their sports participation because of their groin pain. Inclusion criteria included symptoms for at least 3 months and having received at least 10 weeks of active physiotherapy aimed at strength and coordination of the muscles acting on the pelvis, in particular the adductor muscles. Exclusion criteria were a history of previous surgery in the groin area or other causes for chronic groin pain diagnosed by physical examination or imaging (isolated adductor strain/tendinopathy, femoroacetabular impingement, intra-abdominal causes, nerve entrapment, genitourinary causes). The study was approved by the medical ethics committee of the Medical Center Leeuwarden (April 15, 2013; no. nWMO 9). Written informed consent was obtained from all participants.

### Procedures

Physical examination was performed by a sports medicine physician and a surgeon, both experienced in groin pathology, according to the guidelines proposed by Hölmich et al. [22].

Additional imaging (ultrasound, X-ray of the pelvis/hips and SPECT-CT scan) was performed to rule out other pathology causative of groin pain.

Surgical treatment consisted of endoscopic totally extraperitoneal (TEP) hernia repair. Surgery was performed under general anesthesia. A 10 x 15 cm lightweight polypropylene mesh (Ultrapro, Ethicon, a Johnson & Johnson company, Amersfoort, The Netherlands) was placed in a tension free manner without fixation. All operations were performed by two surgeons with extensive experience in endoscopic hernia surgery. Intra-operative findings were recorded. In case of a true inguinal hernia, classification according to Nyhus [23] was used. All patients were discharged from the hospital within 24 hours from surgery.

Patients were advised to take paracetamol in combination with non-steroidal anti-inflammatory drugs (NSAID’s) for the first postoperative days for pain management. No restrictions were given except for no strenuous activities in the first week postoperatively. Early return to sport was promoted. A (care as usual) rehabilitation program under supervision of their physiotherapist was advised.

### Follow-up

Patients were contacted by telephone two weeks after surgery. The main purpose of this contact was to evaluate complaints, and to evaluate the rehabilitation program and return to sport. Resumption to sport activity was recorded in their electronic medical records.

The athletes perception about their groin disability was assessed preoperatively and 6 weeks postoperatively by means of the Hip and Groin Outcome Score (HAGOS). Six weeks postoperatively, the patients received the HAGOS by mail.

The HAGOS [21] was developed as a disease-specific health questionnaire, specifically for young people who are suffering from hip and/or groin complaints. The HAGOS was developed and validated according to the Consensus-Based Standards for the Selection of Health Status Measurement Instruments (COSMIN) checklist [24]. The HAGOS consists of 36 items grouped into six dimensions (subscales), assessing: Symptoms, Pain, Physical function in daily living, Physical function in Sport and Recreation, Participation in Physical Activities and Hip and/or groin-related Quality of life. All questions are rated on a 5-point Likert scale, ranging from 0 (representing no symptoms) to 4 (representing extreme symptoms). After completion, each subscale is summed and transformed to a scale ranging from 0 to 100, in which a higher score refers to a better function. Missing data were treated according to the guidelines proposed bij Thorborg et al. [21]. In this study the Dutch version of the HAGOS was used [25], which was also shown to be valid and reliable.

### Statistical analysis

All statistical analyses were performed using IBM SPSS Statistics for Windows (Version 24.0, Armonk, NY: IBM Corp USA). Paired samples T-tests were used to analyze differences in scores on the HAGOS, measured pre-operatively and six weeks after surgery. To evaluate possible differences between patients who did or did not fill in the HAGOS at six weeks following surgery, a non-response analysis was performed to assess possible differences in terms of age, gender, procedure type and pre-operative scores on the HAGOS.

Bootstrap procedures were used for estimating bias and obtaining confidence intervals [26]. Bootstrap procedures were used to mimic the process of obtaining new data sets by sampling with replacement from the observed dataset. Bootstrapping procedures were used (5000 replications of the trail data) to estimate the 95% confidence intervals of the difference in HAGOS subscales before and after surgery. A p-value <0.05 was considered to indicate statistical significance.

## Results

### Descriptive statistics

**Fig 1:**
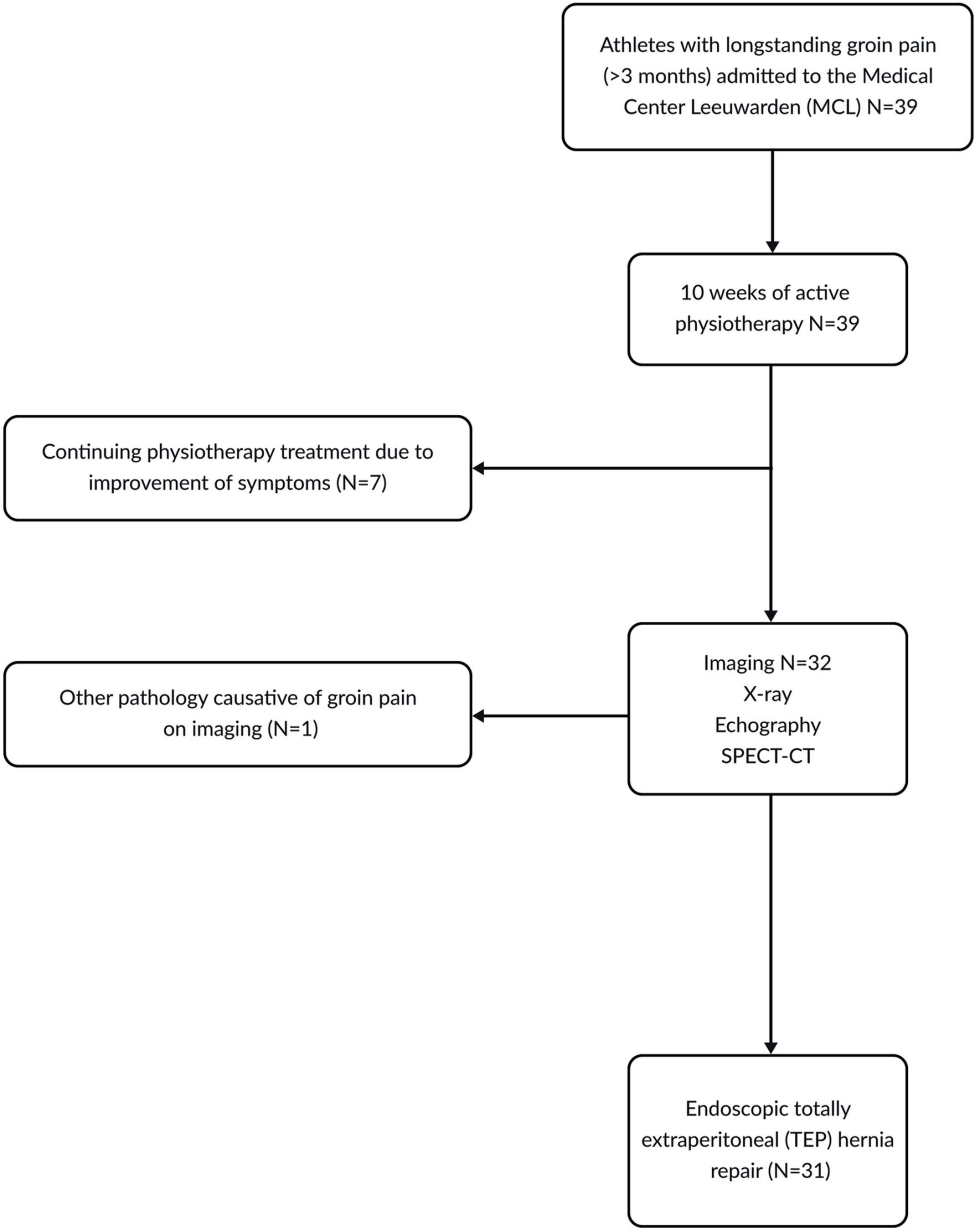
Flow chart for inclusion procedure of patients. Abbreviations: SPECT-CT, single photon-emission computed tomography and CT.

Between 2013 and 2017, 39 patients with groin pain were considered for inclusion in the study. The flow chart of the inclusion procedure of respondents is presented in Fig 1. Seven patients continued physiotherapy due to improvement of symptoms, and one patient was excluded because of clear signs of adductor tendinopathy on the symptomatic side on imaging. Eventually, 31 patients underwent TEP and were included in the study. The study group consisted of 25 soccer players (81%), 4 running athletes (13%) and 2 gymnastics athletes (7%). The demographic characteristics of the patients included in the study are shown in Table 1.

**Table 1:** Baseline characteristics of the patients who were operated with the totally extraperitoneal (TEP) endoscopic inguinal hernia repair technique.

Characteristics	(N = 31) ^a^
Age (years)	27.6 (8.9)
Gender	
Male	31 (100%)
Marital status (N = 30)	
Single	15 (50%)
With partner	3 (10%)
With partner and children	8 (27%)
With children	1 (3%)
With parents	3 (10%)
Educational level (N = 30)	
Elementary school	1 (3%)
High school	9 (30%)
College	10 (33%)
Bachelor’s degree or higher	10 (33%)
Procedure type	
Unilateral	23 (74%)
Bilateral	8 (26%)

^a^ Values, except for age, are given as N (%). Age is given as mean (SD).

### Physical examination and imaging modalities

The findings of physical examination and imaging are presented in Table 2. All patients reported persistent groin pain during sport activities. The pain was unilateral in 23 patients (74%) and bilateral in 8 patients (26%). At physical examination local tenderness over de external and internal ring was found in 25 patients (81%). Also pain at the origin of the adductor muscles 14 (45%) and local tenderness over the pubic tubercle 13 (42%) was a common finding at physical examination. In all patients, no abnormalities were seen on ultrasonographies and x-ray of the pelvis. Of the 31 ultrasonography’s and x-rays of the pelvis. SPECT-CT scans of 13 patients (42%) showed focal increased uptake at the insertion of the adductor muscles at the anteroinferior aspect of the pubic symphysis with no structural abnormality.

**Table 2:** Findings at physical examination and imaging of patients with inguinal disruption.

	(N = 31)
Pain and/or dilatation over the external ring with no obvious hernia evident	25 (81%)
Tenderness over the deep inguinal ring	25 (81%)
Tenderness at the origin of the adductor tendons	14 (45%)
Local tenderness over the pubic tubercle	13 (42%)
Ultrasonography, no abnormalities	31 (100%)
Radiograph of the pelvis, no abnormalities	31 (100%)
SPECT-CT, Increased uptake at symphysis pubis	13 (42%)

Values are given as the number of patients.

### Intra-operative findings

Of the 31 TEP hernia repairs that were performed, 23 were unilateral procedures and 8 bilateral procedures. A true small inguinal hernia was found in 5 patients (16%) and these were all indirect hernias, type 2 according to the Nyhus classification. One lipoma was found near the internal inguinal ring (3%). No other abnormalities were observed during surgery.

### Follow-up after surgery

All wounds healed within two weeks after surgery and no surgical complications occurred. Of the 31 participants, 20 (65%) filled in the HAGOS questionnaire six weeks after surgery. A non-response analysis showed no difference in demographic factors, symptoms and scores of the HAGOS subscales between responders and non-responders.

After six weeks a significant improvement (p-value <0.05) was seen in every subscale of the HAGOS (Table 3). A significant improvement was seen in both the original data and the bootstrapped data. Within six weeks of surgery 26 patients (84%) returned to sports activities with no or less groin pain.

**Table 3:** Bootstrap for paired samples test of HAGOS subscales, based on 5000 bootstrapped samples.

Reliability (n = 20) HAGOS	Preoperative measurement mean (SD)	Six-week measurement mean (SD)	Mean difference (95% CI)	Sig. (2-tailed)
Symptoms	62.9 (14.7)	72.5 (14.1)	-9.6 (-15.1, -4.6)	.003
Pain	72.1 (15.3)	83.8 (13.2)	-11,7 (-20.4, -2.9)	.02
ADL	74.2 (16.3)	87.8 (13.2)	-13.6 (-21.9, -5.6)	.009
Sport/Rec	41.0 (20.1)	65.8 (23.5)	-24.8 (-37.7, -11.3)	.01
PA	19.7 (27.1)	40.1 (36.2)	-20.4 (-34.2, -7.9)	.03
QOL	34.5 (14.1)	56.3 (26.8)	-21.7 (-32.0, -12.5)	.001

Abbreviations: HAGOS: Hip and Groin Outcome Score; ADL, Physical function in daily living; Sport/Rec, Physical function in Sport and Recreation; PA, Participation in Physical Activities; QOL, Hip and or/groin-related Quality of Life.

## Discussion

The results of this prospective cohort study demonstrate clearly that reinforcing the posterior wall with a lightweight polypropylene mesh through endoscopic totally extraperitoneal (TEP) hernia repair is an effective treatment for athletes with inguinal disruption who are unresponsive to conservative treatment. It offers pain relief, improvement in physical function and a fast return to sports activity in the great majority of patients only six weeks after surgery.

Tenderness over the external and internal inguinal ring (81%) was the most common finding during physical examination. These findings are in line with those of van Veen et al. [14], who described local tenderness over the inguinal ring in 88% of the patients diagnosed with ‘Sportman’s hernia’. Tenderness over the pubic tubercle and adductor muscles were also a common finding in our study population. These clinical findings can exist alone or in combination. Of athletes with longstanding groin pain, up to 90 percent are found to have multiple coexisting pathologie [12,27]. The key challenge is determining which of these pathologies is causing groin pain.

In the present study imaging modalities (ultrasound, X-pelvis and SPECT-CT) were used to rule out other causes of groin pain. Despite normal ultrasonography, 5 small inguinal hernias were found peroperatively. Miller et al. [28] showed in 2014 that ultrasonography is a good diagnostic tool for people with clinical signs of an inguinal hernia, but it shows poor sensitivity in patients with a possible occult hernia at physical examination. SPECT-CT showed increased uptake of the pubic bone and symphysis in 13 (42%) of the patients. Abnormal isotope uptake in bone scans at the pubic symphysis represents an increased rate of bone remodeling and is indicative of osteitis pubis. Some authors believe that osteitis pubis should be distinguished from inguinal disruption [17,29]. However, radiological signs of osteitis pubis can also occur in asymptomatic athletes [29]. We agree, according to Paajanen et al. [6] that osteitis pubis should not be used for operative decision making.

Overall, in six (19%) patients abnormalities of the posterior wall were observed per-operatively which consisted of 5 small indirect inguinal hernias and 1 lipoma in the inguinal canal. These results are in line with the per-operative findings reported by authors in the operative management of chronic groin pain in athletes [5,20,30].

To our knowledge only one study [20] used a valid and reliable patient reported outcome measure, which was also the HAGOS to assess physical function and health-related quality of life in athletes who were surgically treated because of groin complaints. They reported a significant improvement on all subscales of the HAGOS, three months after surgery. The largest improvements were seen, as in the current study, in the subscales Physical function in Sport and Recreation, Participation in Physical Activities and Hip and/or groin-related Quality of life. However, to determine if the improvement seen in all subscales of the HAGOS are acceptable, depends on what changes are on the HAGOS are considered important to patients. Improvements in scores should be greater than the Minimally Clinically Important Difference (MCID) to distinguish clinically important difference from statistically significant difference [31]. The MCID of the HAGOS was determined in a study by Kemp et al. [32]. The differences showed in this study on all the subscales of the HAGOS were all greater than or equal to the MCID. Hence, the differences in scores were not only a statistical significant but also clinical important to the patients. A total of 84% of athletes returned to sport activities within 6 weeks. These results are in line with those reported by Van Veen et al. [14].

A strength of our study is that all patients were assessed using the HAGOS before and after surgical treatment. A total of 20 HAGOS questionnaires (65%) were returned. Selection bias may be indicated due to non-responders, making it uncertain if the sample reflects the total study population. However, a non-response analysis showed no differences between responders and non-responders, in terms of age, gender, procedure type and pre-operative scores on the HAGOS.

In general, the generalizability of study results depends on the extent to which the sample data is representative of the total study population. A way to increase the generalizability of study results is the use of bootstrapping methods in order to give a more precise estimation of the outcome population parameter. Regarding the present study, the relatively small sample size at 6 weeks after the operation may be regarded as a limitation of the generalizability of our study results. We therefore used bootstrapping methods to calculate (bootstrapped) confidence intervals of the differences in HAGOS subscales before and after surgery. A significant improvement in scores of the HAGOS was seen in both the original (non-bootstrapped) data and the bootstrapped data.

The lack of MRI may be considered a limitation of this study. Pelvic MRI is often used as diagnostic imaging to visualize pathology in athletes. However, as MRI is based on structural changes in the groin area, it is limited with regard to determine the lesions responsible for the groin pain. Positive MRI findings are often observed in athletes with and without groin pain and can be caused by degenerative changes as well as changes related to exertion [33]. Furthermore, pathology with clinical significance does not always appear as structural change. Hybrid imaging using SPECT-CT allow the direct fusion of morphologic information and functional information. SPECT-CT was utilized to rule out other pathology causative of groin pain. SPECT-CT showed at least comparable diagnostic performance to MRI in prior studies in patients with hand and wrist lesions [34] and also in patients with ankle and foot pain [35].

The lack of a control group (i.e., conservatively treated patients) might be considered a limitation of this study. However, it is difficult to find an adequate control group for surgery as most patients already have tried conservative and alternative treatments for a long time (at least 10 weeks) without any effect. If conservative measures have failed, surgery is provided as an option. Patients serve as their own controls since they are not responding to various conservative treatments. A sham-designed study could be an alternative. However, to perform a surgical procedure that could cause harm without offering a compensating beneficial effect poses ethical problems [36].

Another possible limitation of our study is that no fully standardized post-operative rehabilitation regimen was used. Patients underwent care as usual for their postoperative rehabilitation, which consisted of rehabilitation under supervision of a physiotherapist, chosen by the patient. In general most athletes are able to return to sport 2–6 weeks after laparoscopic surgery [37]. Use of a standardized rehabilitation protocol might accelerate recovery and return to play time.

## Conclusions

Athletes who suffer from chronic groin pain that is caused by inguinal disruption, should be offered a surgical TEP repair when conservative treatment does not alleviate the complaints. This study showed that reinforcement of the posterior wall with a lightweight polypropylene mesh shows good clinical results and improvement of functional status and health-related quality of life of athletes with inguinal disruption.

## Supporting information

S1 Dataset (SAV)
